# Supplementary material for: The microbiome biomarkers of pregnant women’s vaginal area predict preterm prelabor rupture in Western China
Source: Front Cell Infect Microbiol. 2024 Oct 31;14:1471027. doi: 10.3389/fcimb.2024.1471027 (PMC11560878; doi:10.3389/fcimb.2024.1471027)
Supplement: Supplementary file 1 [file DataSheet1.zip › compare_1/Community/Pieplot/genus/group/P.genus.pieplot.pdf]

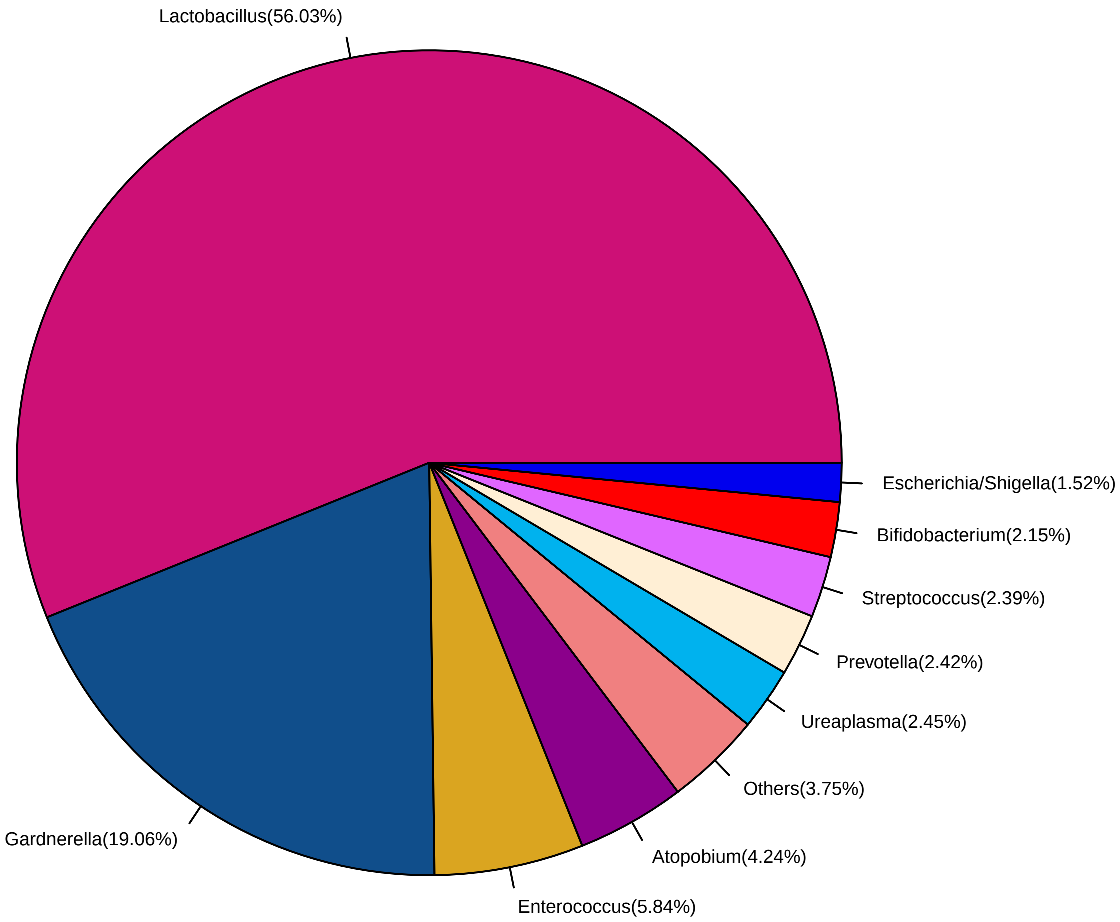
Lactobacillus(56.03%)
Escherichia/Shigella(1.52%)
Bifidobacterium(2.15%)
Streptococcus(2.39%)
Prevotella(2.42%)
Ureaplasma(2.45%)
Others(3.75%)
Atopobium(4.24%)
Enterococcus(5.84%)
Gardnerella(19.06%)
